# Aging Effects on Absolute and Relative Estrogen Receptor Variant Gene Expression Levels in Male Versus Female Rat Ventromedial Hypothalamic Nucleus Growth Hormone-Releasing Hormone Neurons

**DOI:** 10.31083/JIN38142

**Published:** 2025-06-23

**Authors:** Rami Shrestha, Subash Sapkota, Karen P. Briski

**Affiliations:** 1 School of Basic Pharmaceutical and Toxicological Sciences, College of Pharmacy, University of Louisiana Monroe, Monroe, LA 71201, USA

**Keywords:** estrogen receptor-alpha, G protein-coupled estrogen receptor-1, insulin-induced hypoglycemia, Ghrh, sex differences

## Abstract

**Background:**

Aging alters estrogen receptor (ER) expression in distinctive hypothalamic loci, but information regarding potential adjustments in estradiol receptivity at the individual neuron population level remains incomplete. Estradiol controls glucostasis by action on ventromedial hypothalamic nucleus (VMN) targets. VMN growth hormone-releasing hormone (Ghrh) neurons exhibit sex-dimorphic ER variant and counterregulatory transmitter gene profiles in young adult rats.

**Methods:**

Combinatory single-cell laser-catapult-microdissection/multiplex qPCR analyses was used to investigate whether aging changes nuclear versus cytoplasmic *ER* gene expression according to sex.

**Results:**

Ghrh neuron *ER-alpha* and G-protein-coupled estrogen receptor-1 (*GPER*) transcription was decreased in old versus young rats of each sex. Old animals lacked ER-alpha transcriptional reactivity to hypoglycemia, indicative of age-associated loss of response. Hypoglycemia had divergent effects on *ER-beta* transcription, with no effect found in old males versus an inhibitory effect in old female rats. Hypoglycemic inhibition of Ghrh neuron *GPER* gene expression in old male and female rats was similar to that which occurred in corresponding young animals. *Ghrh* gene silencing identified age-related loss of neuropeptide modulatory regulation of *ER* gene transcription. *Ghrh* signaling inhibited eu- and hypoglycemic Ghrh neuron *aromatase*/*CYP19A1* mRNA profiles in old male and female rats; in each sex, this gene transcript was refractory to hypoglycemia regardless of age.

**Conclusions:**

VMN Ghrh neuron neuroestradiol production may be up-regulated with age, but cellular sensitivity to this local steroid signal may differ between young and old rats due to differences in ER variant expression. Further research is warranted to examine how potential age-associated modifications in absolute and proportionate signaling by distinctive ER may affect Ghrh neuron glucose-regulatory neurotransmission in male versus female rats.

## 1. Introduction

The steroid hormone estradiol controls brain function by activating classical nuclear [estrogen receptor-alpha (*ERα*); estrogen receptor-beta (*ERβ*)] and membrane [G-protein-coupled ER (*GPER/GPR30*)] ERs. Estradiol acts by ER-dependent mechanisms to exert critical organizational, neurotrophic, regulatory, and neuroprotective actions over the lifespan [1–7]. During central nervous system (CNS) differentiation, ER stimulation causes gender-imprinting of distinct forebrain loci, resulting in sex-dimorphic cytoarchitectural composition and estradiol regulation of function of the anteroventral periventricular nucleus, medial preoptic area, medial amygdaloid nucleus, stria terminalis bed nucleus, and ventromedial hypothalamic nucleus (VMN) [8–14]. The ER-abundant VMN imposes sex-contingent regulation of arousal, maternal, sexual, aggressive, and defensive behaviors; energy balance; autonomic outflow; and thermogenesis [15–22]. Estradiol shapes body-wide glucose homeostasis [23–27]. The VMN is a foremost sensory and integrative component of the brain glucose regulatory network [21,28] and an important substrate for estradiol control of glycemic and counterregulatory neurochemical profiles [29–31].

Aging has broad-ranging effects on somatic and homeostatic bodily functions, including those governed by ER-regulated neural outflow [32–35]. Indeed, the notion that aging may alter brain cell receptivity to estradiol has garnered ample, justifiable attention. Available studies provide valuable documentation of hypothalamic loci in which age-associated changes in ER expression occur [36–40]. Nevertheless, the unique cyto- and chemo-architectural heterogeneity of each structure accentuates the necessity for high-neuroanatomical resolution analytical techniques to determine if and how aging may affect estradiol receptivity of neurons of characterized functionality. Analysis of brain tissue samples corresponding to whole brain regions, nuclei, or areas poses a risk that averaged endpoint values may mask or obscure unique responses of individual neuron populations. Type 1 diabetes mellitus (T1DM) is a pervasive health concern in the elderly as acute and long-term complications of this condition exacerbate patient morbidity and mortality [41–43]. Older diabetic patients face an elevated risk of hypoglycemia-associated brain injury as counteractive hormone outflow and neurogenic awareness are impaired with age [44–49]. There is an obvious need to identify the mechanism(s) that elicits aging-related counterregulatory collapse. VMN structure is altered by aging in each sex [50]; yet it is unclear if or how age affects ER expression in VMN neurons that engage in neural regulation of glucose homeostasis, and if estradiol-induced changes in cellular function are responsible for these changes.

The VMN functions to assimilate metabolic, hormonal, and neurotransmitter cues to shape glucose counterregulatory responses. Recent studies suggest that estradiol-sensitive dorsomedial VMN (VMNdm) growth hormone-releasing hormone (Ghrh) neurons are a common substrate for these diverse regulatory stimuli. Our work shows that this nerve cell type expresses mRNA that encodes the VMN-exclusive [51,52] metabolic transcription factor steroidogenic factor-1 (SF-1) [53], which is implicated in regulation of systemic energy and glucose balance [54–59]. In addition, *SF-1* gene expression displays a differential response to insulin-induced hypoglycemia (IIH) [60]. VMNdm Ghrh neurons also express hypoglycemia-responsive genes that encode metabolic-sensory biomarkers (i.e., the glucose sensor glucokinase and catalytic subunit of the ultra-sensitive energy sensor 5′-AMP-activated protein kinase [61]) and, in addition to *Ghrh* mRNA, transcripts for biosynthetic enzyme surrogates of characterized counterregulatory-constraining (*γ*-aminobutyric acid) or -enhancing (glutamate; nitric oxide) neurochemicals [60]. Evidence for concurrent expression of neurotransmitters of unique chemical structure, spatial, and temporal profiles that exert characteristic control of counterregulatory hormone secretion supports the provision of complex, coordinated multi-modal signal input by VMNdm Ghrh neurons to the glucostatic neural network.

Developmental organization of the sex-dimorphic VMN results in sex-specific estradiol regulation of VMN functions. VMNdm Ghrh neurons from young adult male and female express *ERα*, *ERβ*, and *GPER* mRNAs, but these ER variants exhibit sex-contingent Ghrh receptor (Ghrh-R)-dependent transcriptional responses to hypoglycemia [60]. Current research made use of *in vivo* gene silencing tools and combinative *in situ* immunocytochemistry, single-cell laser-catapult-microdissection, and single-cell multiplex qPCR techniques to examine the premise that aging may alter eu- and/or hypoglycemic patterns of VMNdm Ghrh neuron *ER* variant gene expression, and that such changes may correlate with age-associated adjustments in Ghrh-R control of distinctive *ER* mRNA profiles. The study design here incorporated animals of each sex to advance U.S. National Institutes of Health policy interest in evaluation of sex as an important biological variable. Firstly, present data depict and compare between sexes aging effects on the magnitude of baseline or IIH-associated patterns of expression of individual VMNdm Ghrh neuron *ER* mRNAs. A emerging study supports the intriguing prospect that age-associated shifts in ER*α*/ER*β* ratio may affect gene transcription and other nerve cell functions [62]. Therefore, age impact on the ratio of VMNdm Ghrh nerve cell *ERα*, *ERβ*, and *GPER* mRNA content during normoand hypoglycemia was also examined in rats of each sex.

Central ERs are activated by ligand that is synthesized in the ovary or within the brain, where testosterone is metabolized to neuroestradiol by aromatase (CYP19A1) enzyme action [63,64]. A small number of neural structures, including the VMN, are characterized by relatively high CYP19A1 gene and protein expression and enzyme activity profiles compared to other brain loci [65–69]. A recent study provides evidence to infer that VMN neuroestradiol influences neural governance of glucose counterregulation [70]. The cellular source(s) of neuroestradiol produced in the VMN or elsewhere remains to be characterized. The present study investigated the corollary hypothesis that VMNdm Ghrh neurons express *CYP19A1*, the gene that encodes CYP19A1 protein, and that eu- and/or hypoglycemia-associated transcription of this gene may be affected by age in one or both sexes.

## 2. Materials and Methods

### 2.1 Animals

Young adult (2–3 months of age) and old (11–12 months of age) male and female Sprague Dawley rats were accommodated communally by sex in shoe-box cages (2–3 animals per cage), under a 14-hr light: 10-hr dark cycle (lights on at 05.00 hr). Animals were habituated to daily handling before initiation of study procedures and had ad-libitum access to standard laboratory chow (prod. no. Harlan Teklad LM-485; Harlan Industries, Madison, WI, USA) and tap water. Study protocols and procedures were performed in compliance with the NIH Guide for Care and Use of Laboratory Animals, 8th Edition, under approval of the university Institutional Animal Care and Use Committee.

### 2.2 Research Design

On Study Day 1, old rats of each sex were randomly assigned to four treatment groups (n = 4 animals/sex/treatment group). Young male (n = 4) and female (n = 4) rats were designated as age-associated controls. Animals were anesthetized by intraperitoneal injection of 9.0 mg ketamine (056344, Covetrus North America, Dublin, OH, USA)/1.0 mg xylazine (061035, Covetrus North America)/0.1 mL/100 g *bw* before infusion of *Ghrh* siRNA (500 pmol; Accell siRNA rat Ghrh, set of 4; A-089046–16-0010; Horizon, Cambridge, UK) or scramble (*SCR*) siRNA (500 pmol; Accell Control Pool Non-Targeting; D-001910–10-20; Horizon) to the bilateral VMN (coordinates: −2.5 mm posterior to bregma, 0.6 mm lateral to midline, 9.0 mm ventral to skull surface), in a 1.0 μL total volume at a 3.6 μL/min infusion rate; as described [60]. Injections were made using a 33-gauge Neuros syringe (53496; Stoelting Co., Wood Dale, IL, USA), under Neurostar stereotactic Drill & Injection Robot computer control (Neurostar, Tubingen, Germany). This genetic manipulation results in significant suppression of VMNdm Ghrh nerve cell Ghrh mRNA and protein [60]. Post-surgical treatments included subcutaneous(*sc*) ketophen (738764, Zoetis Inc., Kalamazoo, MI, USA) and IM enrofloxacin (00724089018656, Bayer HealthCare LLC, Animal Health Division, Shawnee Mission, KS, USA) injections and topical 0.5% bupivacaine (0409–1163-18, Hospira, Inc., Lake Forest, IL, USA) application to closed incisions. Upon full recovery from anesthesia, animals were transferred to single-occupancy cages. On Study Day 7, male and female rats received a *sc* injection with vehicle (V; sterile diluent; Eli Lilly & Co., Indianapolis, IN, USA) or neutral protamine Hagedorn insulin (INS; 10.0 U/kg *bw*; Eli Lilly & Co. [71]) at 09.00 hr; animals were sacrificed one hr post-injection by rapid decapitation. Individual whole brains were dissected for snap-freezing achieved by immersion in liquid nitrogen-cooled isopentane and storage at −80 °C.

### 2.3 Individual VMNdm Ghrh Neuron Laser-Catapult-Microdissection

Consecutive 10 μm-thick fresh-frozen tissue sections of the VMN were collected between −1.80 to −2.3 mm posterior to bregma and mounted on polyethylene naphthalate membrane-coated slides (415190–9041-000; Carl Zeiss Microscopy LLC, White Plains, NY, USA). Serial sectioning of each brain was initiated rostral to the retrochiasmatic area, at the approximate level of the optic chiasm, and continued until the VMN was reached; sections then cut through the rostral VMN were processed to acquire pure Ghrh nerve cell samples by *in situ* immunocytochemistry/laser-catapult microdissection for gene expression analysis. Several distinctive neurotopographic features were used to verify the rostro-caudal progression of tissue sectioning, including continuity of the third ventricle with lateral ventricles, which occurs at the level of the suprachiasmatic nucleus; derivation of the lateral optic tracts from the midline optic chiasm; and rostro-caudal changes in curvature of the ventral surface of the hypothalamus. VMN sections were fixed with ice-cold acetone (5 min), then blocked (2 h) with 1.5% normal goat serum (S-1000, Vector Laboratories, Burlingame, CA, USA) in Tris-buffered saline (TBS), pH 7.4, supplemented with 0.05% Triton X-100 (T8787, Sigma-Aldrich, St. Louis, MO, USA). Tissues were next incubated (48–72 h; 4 °C) with a rabbit primary antiserum against preproGhrh (PA5–102738, 1:2500; Invitrogen, Waltham, MA, USA), as described [60], followed by a goat anti-rabbit horseradish peroxidase-labeled secondary antibody (1 h; PI-1000, 1:1000; Vector Laboratories). Ghrh immunoreactivity (-ir) was manifested using ImmPACT 3,3′-diaminobenzidine peroxidase substrate kit reagents (SK-4105; Vector Laboratories). Ghrh-ir-positive neurons were individually isolated and removed from tissue sections using a Zeiss P.A.L.M. UV-A microlaser IV system ( Carl Zeiss Microscopy LLC), and collected into an adhesive cap (415190–9181-000; Carl Zeiss Microscopy LLC) holding lysis buffer (4 μL; Single Shot Cell Lysis Kit; 1725080; Bio-Rad, Hercules, CA, USA) for single-cell multiplex quantitative reverse transcription PCR (RT-qPCR) analysis.

### 2.4 Single-Cell Multiplex RT-qPCR Analysis

Complementary DNA (cDNA) synthesis and amplification: For each animal, n = 3 Ghrh-ir neurons, the statistical unit in this study, were collected for single-cell gene expression analysis. Thus, for each treatment group, a total of N = 12 neurons was evaluated for male subjects and N = 12 neurons were analyzed for female subjects. Individual cell lysates were centrifuged (3000 rpm; 4 °C; 5 min) before serial 25 °C (10 min) and 75 °C (5 min) incubations in a Bio-Rad iCyclerQ RT-PCR Detection System (170–8740; Bio-Rad). Sample RNA quantity and purity were verified by NanoDrop spectrophotometry (ND-ONE-W, Thermo Fisher Scientific (ThermoFisherSci.), Waltham, MA, USA). Single-cell mRNA was reverse-transcribed to cDNA by addition of 1.5 μL iScript^™^ Advanced cDNA Synthesis Kit buffer (1725038; Bio-Rad), followed by 46 °C incubation (20 min) and 95 °C denaturation (1 min), as previously described in detail [72–74]. Pre-amplification master mix was prepared by combining SsoAdvanced^™^ Pre-Amp Supermix (1725160; Bio-Rad) with individual target and housekeeping gene Bio-Rad PrimePCR^™^ PreAmp for SYBR Green Assays [ERalpha (*ERα*) (qRnoCID0009588), ESR2/ERbeta (*ERβ*) (qRnoCID0008785), *GPER* (qRnoCED0007818), *CYP19A1* (qRnoCID0007793), *Ghrh* (qRnoCID0007723), and *GAPDH* (qRnoCID0057018; Bio-Rad) with SsoAdvanced^™^ PreAmp Supermix (1725160; Bio-Rad)]. After addition of 9.5 μL pre-amplification master mix, cDNA samples were initially incubated at 95 °C (3 min), then exposed to 20 cycles of consecutive 95 °C (15 sec) and 58 °C (4 min) incubations. RT-qPCR analysis: cDNA samples were diluted with IDTE (185 μL; 11–05-01–05; Integrated DNA Technologies, Inc., Coralville, IA, USA). PCR samples were prepared by combining 2.0 μL cDNA sample with target gene Bio-Rad primers [*ESR1* (0.5 μL; qRnoCID0009588), *ESR2* (0.5 μL; qRnoCID0008785), *GPER* (0.5 μL; qRnoCED0007818), *CYP19A1* (0.5 μL; qRnoCID0007793), *Ghrh* (0.5 μL; qRnoCID0007723), and *GAPDH* (0.5 μL; qRnoCID0057018)] and 5.0 μL iTaq^™^Universal SYBR^®^ Green Supermix (1725121; BioRad), then added to individual wells of hard-shell 384-well PCR plates (HSP3805, Bio-Rad) for analysis in a Bio-Rad CFX384^™^ Touch Real-Time PCR Detection System (CFX384^™^, Bio-Rad). Samples were initially denatured at 95 °C (30 sec), then incubated over 40 cycles of initial 95 °C incubation (3 sec), then 30 sec incubation he at 60 °C for *ESR1*; 59.9 °C for *ESR2*; 59.8 °C for *GPER*; 59.6 °C for *CYP19A1*, 58.5 °C for *Ghrh*, or 57.3 °C for *GAPDH*, respectively. Melt curve analyses were performed to detect primer dimer and nonspecific product formation. Data were normalized relative to *GAPDH* by means of the comparative Ct (2^−ΔΔCt^) method [75].

### 2.5 Statistics

Mean normalized mRNA values for old male and female rats were analyzed by three-way ANOVA and Student Newman Keuls *post-hoc* test. Data from young SCR siRNA/V versus old SCR siRNA/V control groups were evaluated by *t* test. Differences of *p* < 0.05 were regarded as significant. In each figure, statistical differences between individual pairs of treatment groups are denoted by the following symbols: *p < 0.05; **p < 0.01; ***p < 0.001.

## 3. Results

Aging has documented effects on net ER variant mRNA and/protein content in distinct hypothalamic loci but understanding of how age may impact estradiol sensitivity of neurons that operate within the glucostatic neural circuitry is lacking. VMNdm Ghrh neurons likely function to integrate metabolic, endocrine, and neurochemical regulatory cues to shape counterregulatory neurotransmission. Here, single-cell laser-catapult-microdissection/multiplex qPCR analytical tools were applied to investigate whether aging perturbs, in one or both sexes, the magnitude and/or proportional expression of VMNdm Ghrh neuron ER variant mRNAs during eu- or hypoglycemia. Current research also examined the prospect that Ghrh neuromodulatory regulation of VMNdm Ghrh nerve cell *ER* gene transcription may change with aging in male and/or female rats using stereotactic delivery of *in vivo Ghrh* gene silencing tools.

**Fig. 1.**
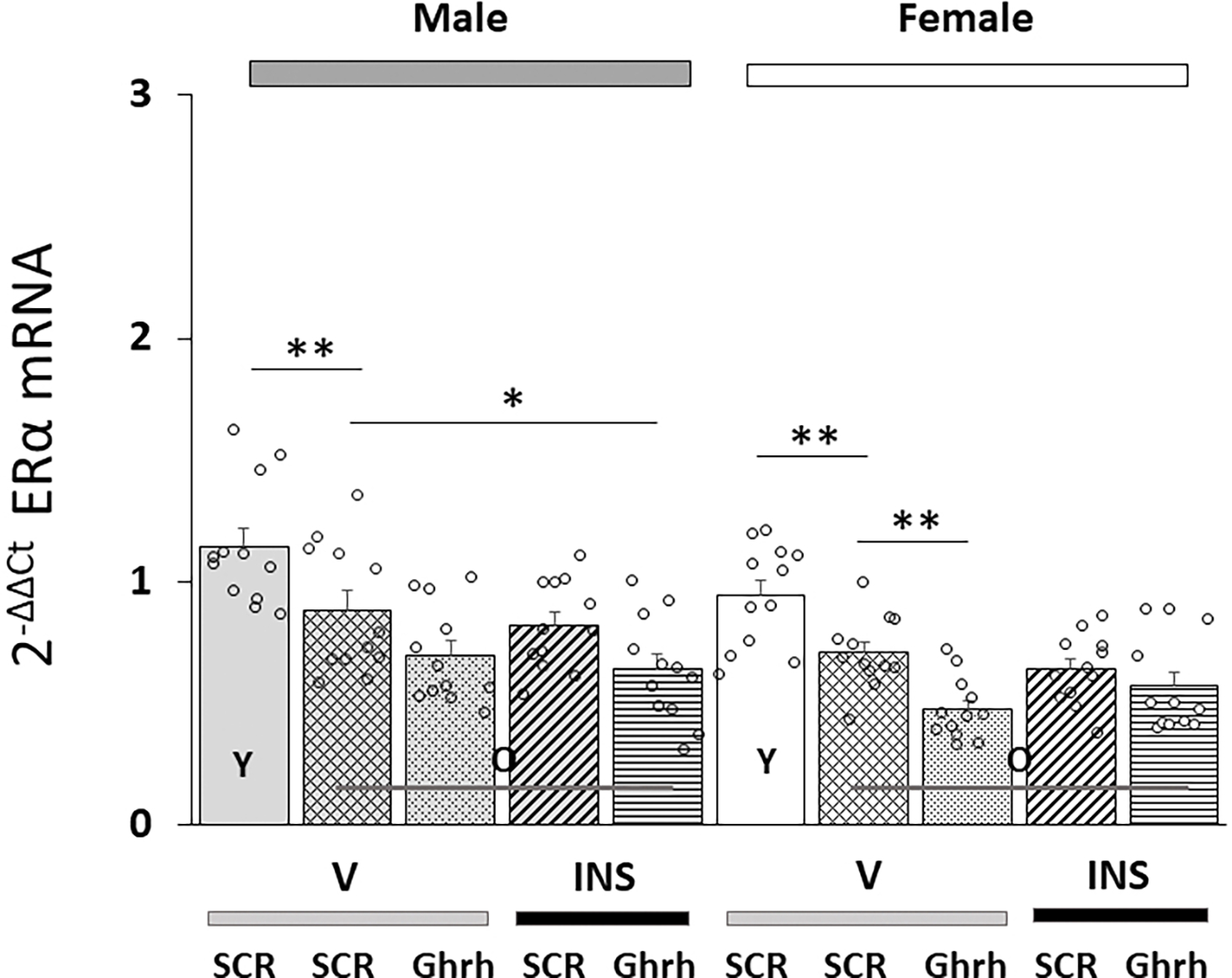
Effects of ventromedial hypothalamic nucleus (VMN) growth hormone-releasing hormone (*Ghrh*) gene knockdown on total estrogen receptor-alpha (*ERα*/*ESR1*) gene expression in old male versus female dorsomedial VMN (VMNdm) Ghrh-immunoreactive (-ir) neurons. Groups of old male and female rats (n = 4 males and n = 4 females per group) were pretreated by bilateral intra-VMN scramble (*SCR*) or *Ghrh* siRNA infusion seven days before subcutaneous (*sc*) injection of vehicle (V) or neutral protamine Hagedorn insulin (INS; 10.0 U/kg *bw*). Young *SCR* siRNA-pretreated, V-injected male and female rats served as controls. Individual brains were collected one hour after injection and cut into 10 micron-thick fresh frozen sections through the VMN; tissues were processed by immunocytochemistry to identify VMNdm Ghrh-ir neurons for single-cell laser-catapult-microdissection/multiplex quantitative real-time polymerase chain reaction (qPCR) analysis. mRNA values were normalized to the housekeeping gene *GAPDH* by the 2^−ΔΔCt^ method [75]. Data depict mean normalized total VMNdm Ghrh neuron *ERα* mRNA measures + S.E.M. for male (five bars, *at left*) and female (five bars, *at right*) rat treatment groups. Old rat treatment groups (identified by the abbreviation ‘O’) are identified as follows: *SCR* siRNA/V (cross-hatched bars; male: gray, n = 12; female: white, n = 1); *Ghrh* siRNA/V (stippled bars; male: gray, n = 12; female: white, n = 12); *SCR* siRNA/INS (diagonal-striped bars; male: gray, n = 12; female: white, n = 12); *Ghrh* siRNA/INS (horizontal-striped bars; male: gray, n = 12; female: white, n = 12). Young male and female treatment groups (indicated by the abbreviation ‘Y’) are represented by solid gray (n = 12) or white (n = 12) bars respectively. For each panel, circles depict individual independent data points. mRNA and protein data were analyzed between old rat treatment groups by three-way ANOVA and Student-Newman-Keuls *post-hoc* test. Data from young *SCR* siRNA/V versus old *SCR* siRNA/V control groups were evaluated by *t* test. Statistical differences between discrete pairs of treatment groups are denoted as follows: *p < 0.05; **p < 0.01.

Fig. 1 (Ref. [75]) illustrates effects of VMN *Ghrh* gene silencing on VMNdm Ghrh nerve cell *ERα*/*ESR1* gene expression in eu- and hypoglycemic aged rats. Data show that baseline *ERα* gene profiles were significantly decreased in old versus young animals of each sex [F_(7,88)_ = 10.88, *p* < 0.001; Sex effect: F_(1.88)_ = 16.22, *p* < 0.001; Treatment effect: F_(1,88)_ = 4.38, *p* = 0.039; Pretreatment effect: F_(1.88)_ = 31.52, *p* < 0.001; Sex/treatment interaction: F_(1,88)_ = 1.03, *p* = 0.313; Sex/pretreatment interaction: F_(1,88)_ = 0.26, *p* = 0.605; Treatment/pretreatment interaction: F_(1,88)_ = 6.43, *p* = 0.013; Sex/pretreatment/treatment interaction: F_(1,44)_ = 0.83, *p* = 0.364]. Results also indicate that baseline *ERα* mRNA levels were suppressed by *Ghrh* gene silencing in old female, but not old male rats. This gene profile was refractory to hypoglycemia in both sexes, and patterns of *ERα* expression following INS injection were unaffected by *Ghrh* siRNA pretreatment.

**Fig. 2.**
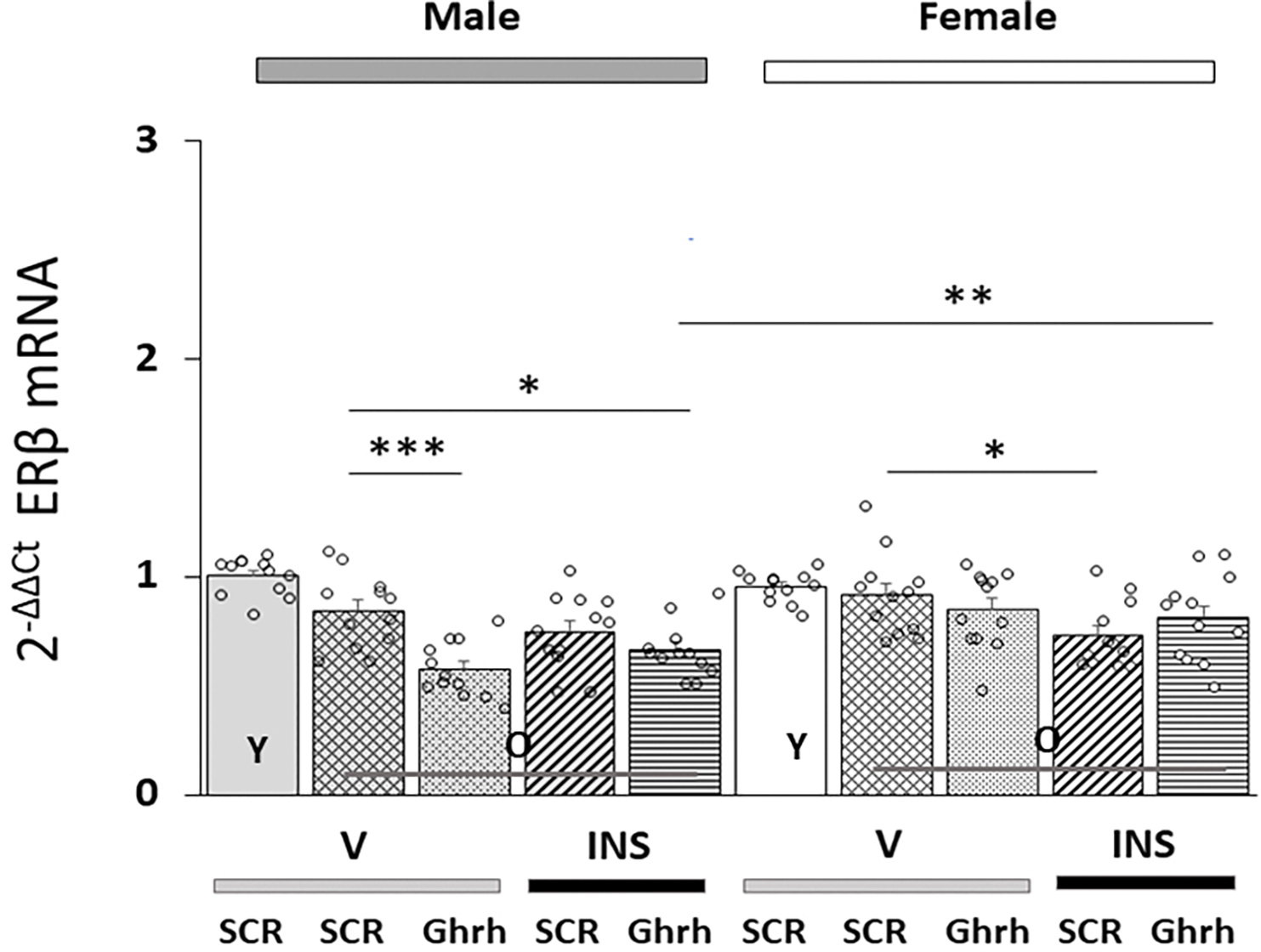
Patterns of VMNdm Ghrh neuron estrogen receptor-beta (*ERβ*/*ESR2*) gene expression in old eu- or hypoglycemic male versus female rats. Results present mean normalized total VMNdm Ghrh nerve cell *ERβ*/V mRNA values + S.E.M. for male (five bars, *at left*) and female (five bars, *at right*) rat treatment groups. Old rat treatment groups, indicated by the abbreviation ‘O’, are identified as follows: *SCR* siRNA/V (cross-hatched bars; male: gray, n = 12; female: white, n = 1); *Ghrh* siRNA/V (stippled bars; male: gray, n = 12; female: white, n = 12); *SCR* siRNA/INS (diagonal-striped bars; male: gray, n = 12; female: white, n = 12); *Ghrh* siRNA/INS (horizontal-striped bars; male: gray, n = 12; female: white, n = 12). Young male and female treatment groups, identified by the abbreviation ‘Y’, are represented by solid gray (n = 12) or white (n = 12) bars respectively. For each panel, circles depict individual independent data points. Normalized mRNA data were analyzed between old rat treatment groups by three-way ANOVA and Student-Newman-Keuls *post-hoc* test Data from young *SCR* siRNA/V versus old *SCR* siRNA/V control groups were evaluated by *t* test. Statistical differences between discrete pairs of treatment groups are denoted as follows: *p < 0.05; **p < 0.01; ***p < 0.001.

Data in Fig. 2 depict patterns of *ERβ*/*ESR2* gene transcription in VMNdm Ghrh neurons collected from old male or female rats pretreated with *Ghrh* versus *SCR* siRNA before *sc* V or INS injection. *ERβ* mRNA levels were not significantly different in old versus young rats of either sex [F_(7,88)_ = 10.43, *p* < 0.001; Sex effect: F_(1.88)_ = 12.19, *p* = 0.001; Treatment effect: F_(1,88)_ = 7.77, *p* = 0.006; Pretreatment effect: F_(1.88)_ = 13.76, *p* < 0.001; Sex/treatment interaction: F_(1,88)_ = 1.78, *p* = 0.185; Sex/pretreatment interaction: F_(1,88)_ = 13.20, *p* < 0.001; Treatment/pretreatment interaction: F_(1,88)_ = 13.41, *p* < 0.001; Sex/pretreatment/treatment interaction: F_(1,44)_ = 0.65, *p* = 0.423]. Baseline *ERβ* gene transcription was decreased in old male, but not old female rats. Hypoglycemia diminished this gene profile in old female, but not old male rats; patterns of *ERβ* gene expression following INS injection were unaffected by prior *Ghrh* siRNA pretreatment.

**Fig. 3.**
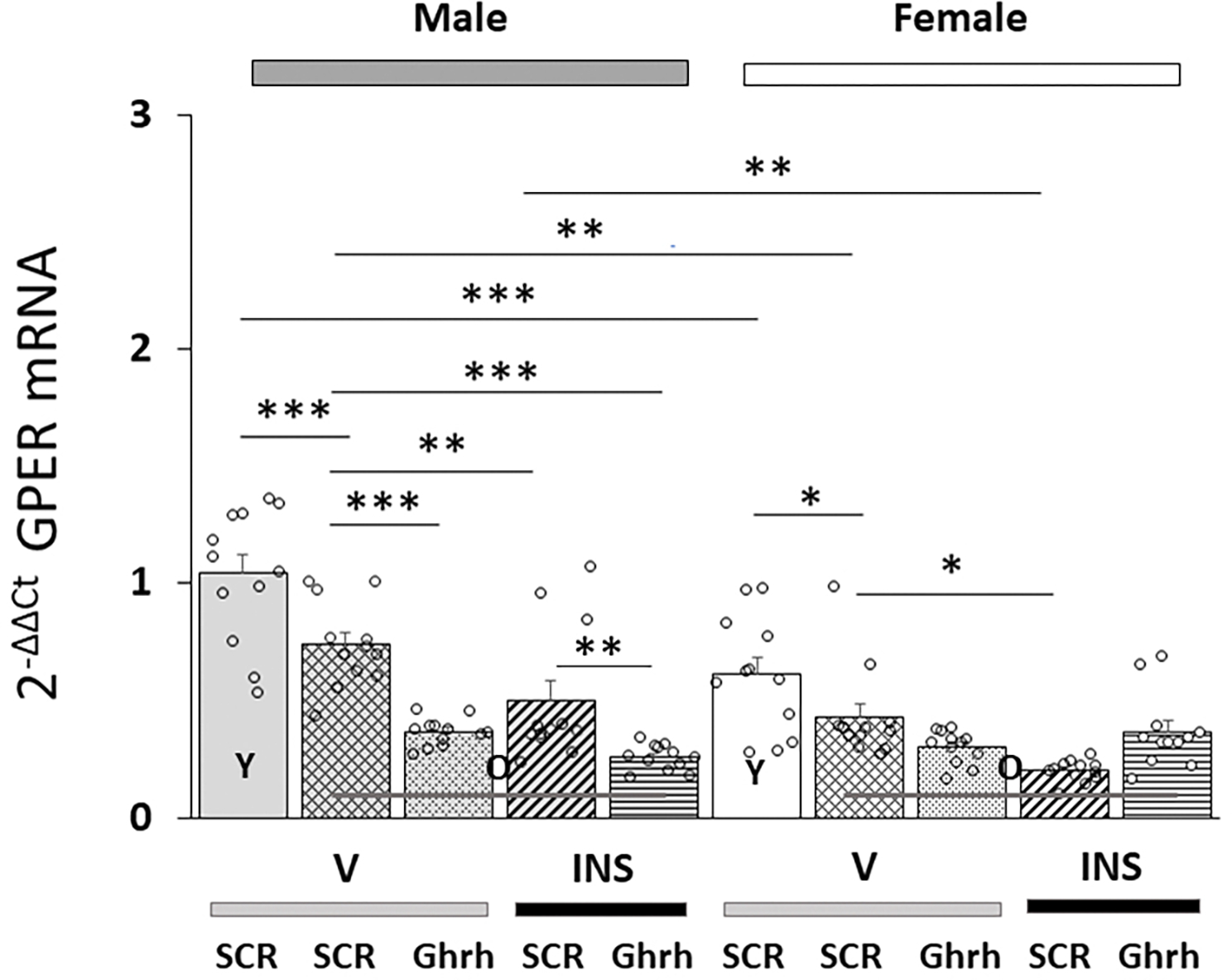
Effects of VMN *Ghrh* Gene Silencing on Eu- and Hypoglycemic Patterns of VMNdm Ghrh neuron G-protein-coupled-estrogen receptor-1 (*GPER*/*GRP31*) mRNA expression in old male versus female rats. Data show mean total *GPER* mRNA values + S.E.M. for male (five bars, *at left*) and female (five bars, *at right*) rat treatment groups. Old rat treatment groups, indicated by the abbreviation ‘O’, are identified as follows: *SCR* siRNA/V (cross-hatched bars; male: gray, n = 12; female: white, n = 1); *Ghrh* siRNA/V (stippled bars; male: gray, n = 12; female: white, n = 12); *SCR* siRNA/INS (diagonal-striped bars; male: gray, n = 12; female: white, n = 12); *Ghrh* siRNA/INS (horizontal-striped bars; male: gray, n = 12; female: white, n = 12). Young male and female treatment groups, indicated by the abbreviation ‘Y’, are represented by solid gray (n = 12) or white (n = 12) bars respectively. For each panel, circles depict individual independent data points. Normalized mRNA data were analyzed between old rat treatment groups by three-way ANOVA and Student-Newman-Keuls *post-hoc* test. Data from young *SCR* siRNA/V versus old *SCR* siRNA/V control groups were evaluated by *t* test. Statistical differences between discrete pairs of treatment groups are denoted as follows: *p < 0.05; **p < 0.01; ***p < 0.001.

Fig. 3 shows effects of VMN *Ghrh* siRNA administration on VMNdm Ghrh nerve cell *GPER* gene expression in eu- or hypoglycemia old male versus female rats. Data indicate that in each sex, baseline *GPER* mRNA content was significantly lower in old versus young animals [F_(7,88)_ = 22.84, *p* < 0.001; Sex effect: F_(1.88)_ = 17.16, *p* < 0.001; Treatment effect: F_(1,88)_ = 24.74, *p* < 0.001; Pretreatment effect: F_(1.88)_ = 28.98, *p* < 0.001; Sex/treatment interaction: F_(1,88)_ = 2.55, *p* = 0.113; Sex/pretreatment interaction: F_(1,88)_ = 22.16, *p* < 0.001; Treatment/pretreatment interaction: F_(1,88)_ = 19.29, *p* < 0.001; Sex/pretreatment/treatment interaction: F_(1,44)_ = 0.46, *p* = 0.501]. Hypoglycemia caused a significant reduction in this gene profiles in old male and female rats. This inhibitory response was exacerbated in old males but was reversed in old females.

**Fig. 4.**
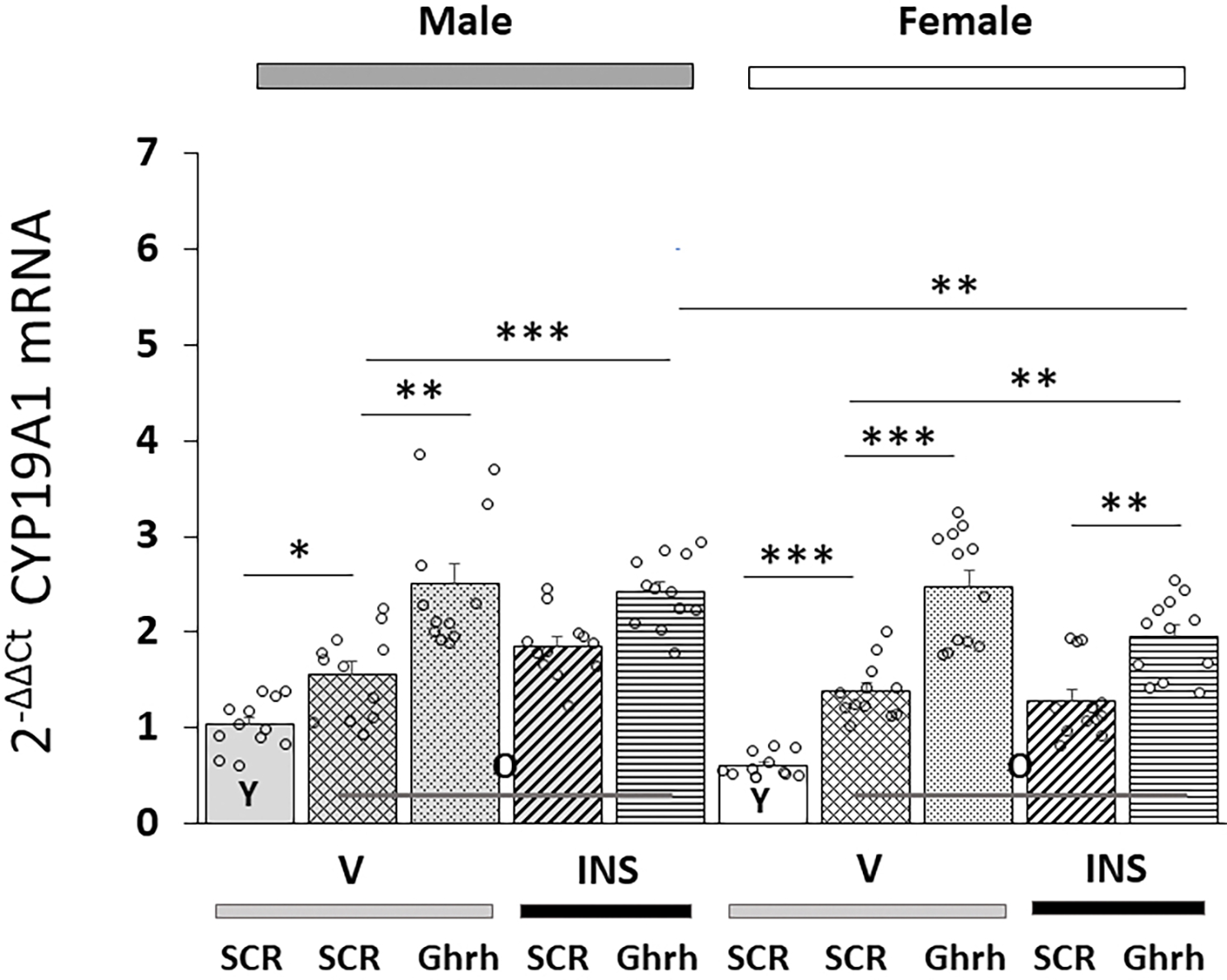
VMNdm Ghrh neuron aromatase (*CYP19A1*) gene expression in old male versus female rat VMNdm Ghrh neurons. Data depict mean total *CYP19A1* mRNA values ± S.E.M. for male (five bars, *at left*) and female (five bars, *at right*) rat treatment groups. Old rat treatment groups, indicated by the abbreviation ‘O’, are identified as follows: *SCR* siRNA/V (cross-hatched bars; male: gray, n = 12; female: white, n = 1); *Ghrh* siRNA/V (stippled bars; male: gray, n = 12; female: white, n = 12); *SCR* siRNA/INS (diagonal-striped bars; male: gray, n = 12; female: white, n = 12); *Ghrh* siRNA/INS (horizontal-striped bars; male: gray, n = 12; female: white, n = 12). Young male and female treatment groups, indicated by the abbreviation ‘Y’, are represented by solid gray (n = 12) or white (n = 12) bars respectively. For each panel, circles depict individual independent data points. Normalized mRNA data were analyzed between old rat treatment groups by three-way ANOVA and Student-Newman-Keuls *post-hoc* test. Data from young *SCR* siRNA/V versus old *SCR* siRNA/V control groups were evaluated by *t* test. Statistical differences between discrete pairs of treatment groups are denoted as follows: *p < 0.05; **p < 0.01; ***p < 0.001.

Patterns of VMNdm Ghrh nerve cell *CYP19A1* gene expression in *Ghrh* versus *SCR* siRNA-pretreated, eu- or hypoglycemic old male and female rats are shown in Fig. 4. Data indicate that this gene profile was significantly up-regulated in old versus young animals of each sex [F_(7,88)_= 25.39, *p* < 0.001; Sex effect: F_(1.88)_= 14.66, *p* < 0.001; Treatment effect: F_(1,88)_ = 0.43, *p* = 0.512; Pretreatment effect: F_(1.88)_= 116.62, *p* < 0.001; Sex/treatment interaction: F_(1,88)_= 3.66, *p* = 0.058; Sex/pretreatment interaction: F_(1,88)_= 0.95, *p* = 0.332; Treatment/pretreatment interaction: F_(1,88)_= 15.96, *p* < 0.001; Sex/pretreatment/treatment interaction: F_(1,44)_= 0.24, *p* = 0.625]. *Ghrh* gene silencing increased *CYP19A1* mRNA levels in VMNdm Ghrh neurons from old male or female rats. Hypoglycemia alone did not modify *CYP19A1* gene transcription; yet *Ghrh* siRNA pretreatment amplified this gene profile in INS-injected old rats of each sex.

**Fig. 5.**
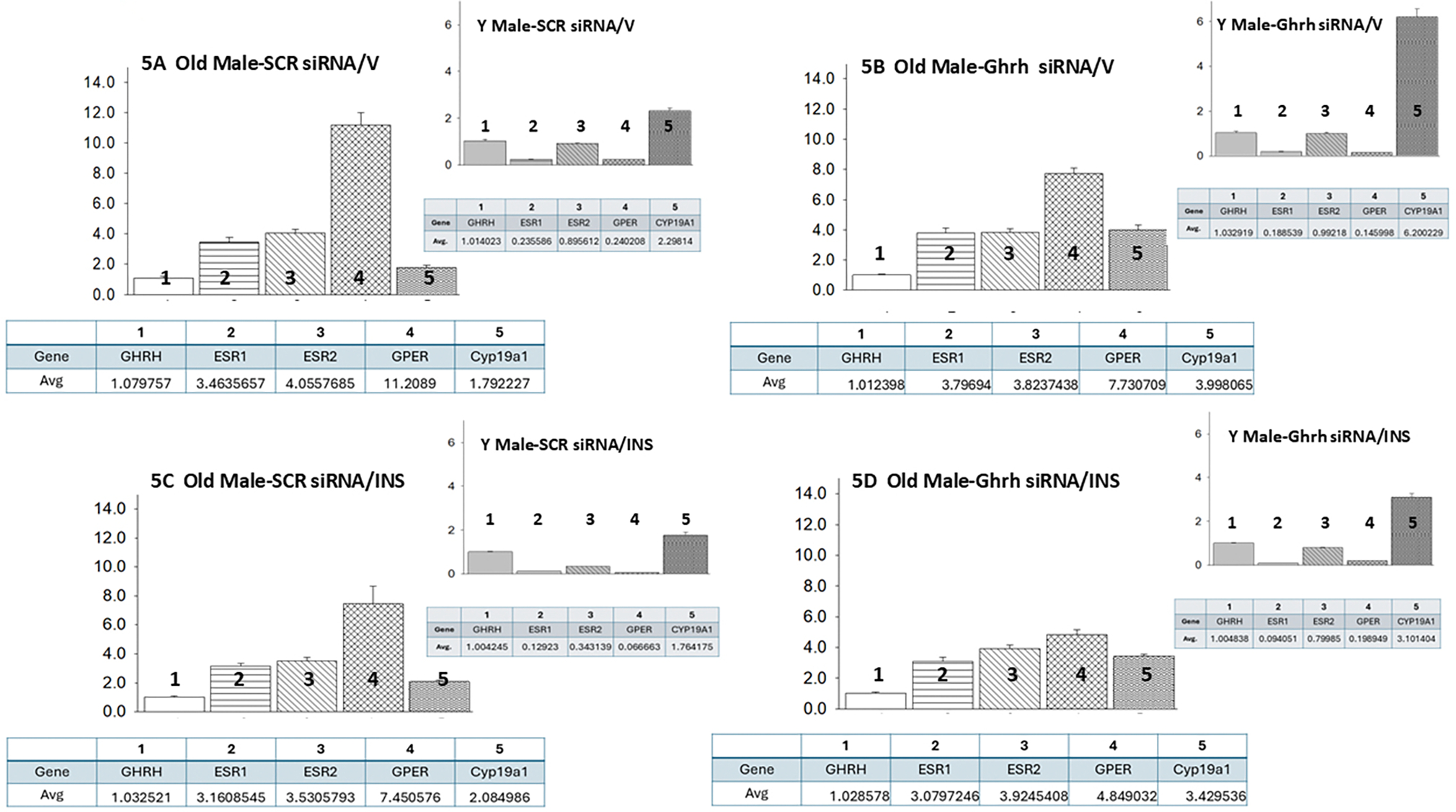
Mean ER variant and *CYP19A1* mRNA relative expression ratios for eu- and hypoglycemic old male rat VMNdm Ghrh/SF-1 neurons. Multiplex single-cell qPCR data acquired from laser-catapult-microdissected Ghrh–ir neurons here were used to establish mean ratios for target gene expression levels relative to *Ghrh* mRNA. Averaged ratio values were derived from n = 12 laser-catapult-microdissected VMNdm Ghrh-ir neurons from old male rats treated as follows: *SCR* siRNA/V (A), *Ghrh* siRNA/V (B), *SCR* siRNA/INS (C), *Ghrh* siRNA/INS (D). In each figure panel, mean relative gene expression data are depicted in graphical (above; bars with accompanying S.E.M.) and tabular (below; top row: gene name; bottom row: average proportionate expression ratio value) formats. The insert to each panel depicts proportionate expression of ER variant and *CYP19A1* transcripts relative to Ghrh in young male rats; these data were mined from the data set from which information on Ghrh gene knockdown effect on absolute target gene expression in young rats was previously reported [60].

Fig. 5 (Ref. [60]) presents VMNdm Ghrh neuron ER variant and *CYP19A1* gene expression ratios for *SCR* versus *Ghrh* siRNA-pretreated *sc* V- (Fig. 5A,B) or INS- (Fig. 5C,D) injected old male rats. Each panel shows average ratios of individual target genes, identified by number, versus *Ghrh* mRNA; ratio values are depicted in graphical (above) and tabular (below) formats. An insert figure in the upper right-hand corner of each panel illustrates expression ratios for VMNdm *Ghrh* neuron target genes for comparable young male rat treatment groups. Data in Fig. 5A show that under euglycemic conditions, this nerve cell type exhibits differential magnitude of ER variant gene transcription, as indicated by dissimilar average ratio values, with *GPER* gene expression evidently predominating compared to nuclear *ER* gene profiles. This gene ratio reflects a change in proportionate transcription from young animals which are characterized by higher relative *ERβ* mRNA levels versus *ERα* and *GPER*. Fig. 5B depicts *Ghrh* gene knockdown effects on individual gene expression ratios and provides numerical notation of change in ratio values versus *SCR* siRNA/V controls. Data infer that this treatment respectively diminishes or augments proportionate *GPER* and *CYP19A1* gene transcription in both old and young male rats. Comparison of VMNdm *Ghrh*/*SF-1* neuron relative target gene expression in hypo- (Fig. 5C) versus euglycemic (Fig. 5A) old male rats indicates that glucose deficiency causes inverse changes to proportionate *GPER* (decreased) and *CYP19A1* (increased) gene expression. Young male rats exhibit a similar negative change in relative *GPER* mRNA profiles, alongside decreased *ERα*, *ERβ*, and *CYP19A1* gene expression. *Ghrh* control of proportional *GPER* and *CYP19A1* transcription is comparable in eu- versus hypoglycemic old male rats, whereas young hypoglycemic male rats exhibit a gain of negative Ghrh control of relative *ERβ* gene expression and switch in direction of control of *GPER* (stimulatory to inhibitory) gene ratio compared to euglycemic animals. Data in Fig. 5 also show that *Ghrh* gene silencing increased *CYP19A1* gene relative expression ratios in old and young eu- or hypoglycemic male rats, whereas hypoglycemia caused divergent changes in this ratio, i.e., increased in old versus decreased in young animals.

**Fig. 6.**
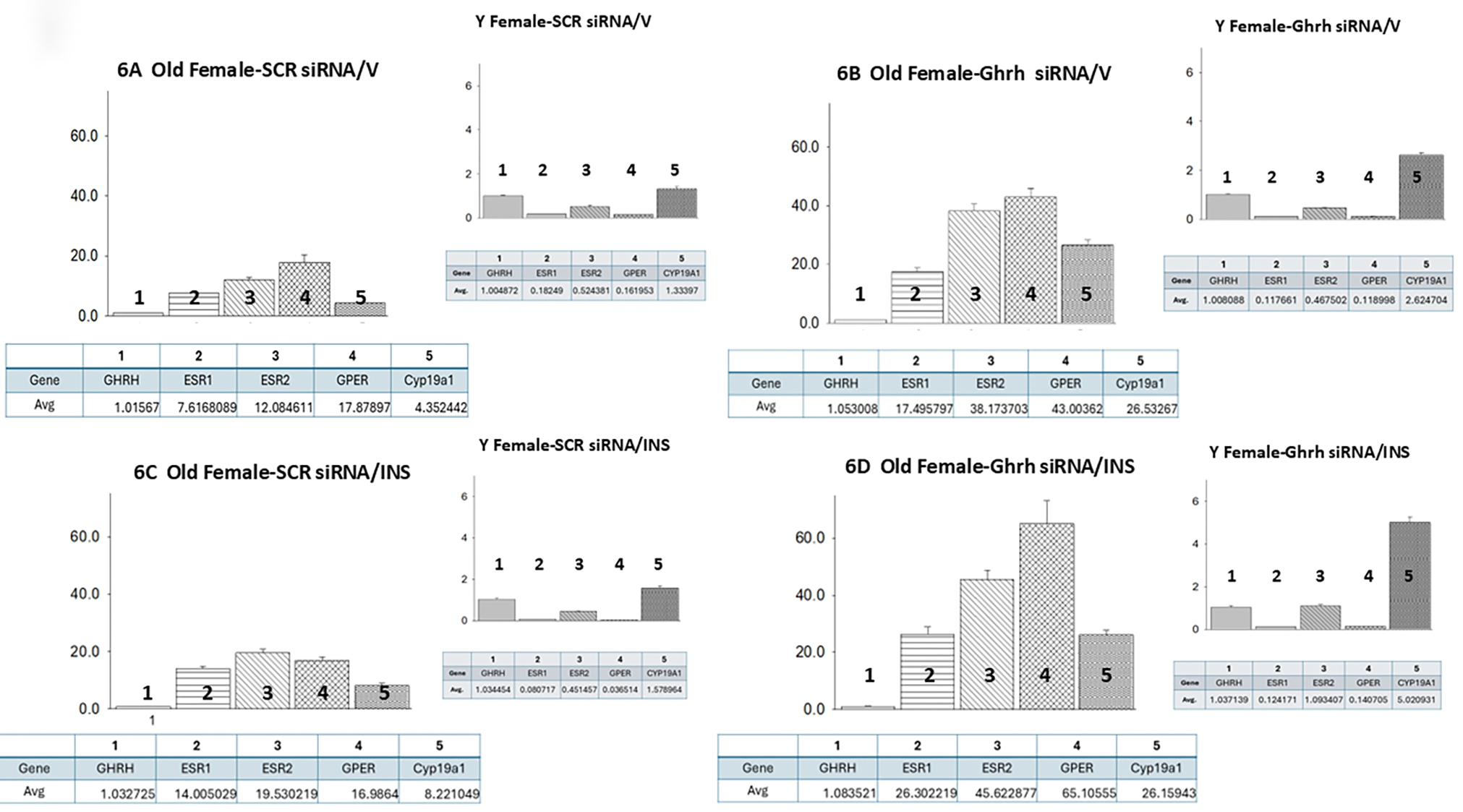
Mean ER variant and *CYP19A1* mRNA relative expression ratios for eu- and hypoglycemic old female rat VMNdm Ghrh/SF-1 neurons. Multiplex single-cell qPCR data acquired from laser-catapult-microdissected Ghrh–ir neurons here were used to establish mean ratios for target gene expression levels relative to *Ghrh* mRNA. Averaged ratio values were derived from n = 12 laser-catapult-microdissected VMNdm Ghrh-ir neurons from old female animals grouped as follows: *SCR* siRNA/V (A), *Ghrh* siRNA/V (B), *SCR* siRNA/INS (C), *Ghrh* siRNA/INS (D). In each figure panel, mean relative gene expression data are depicted in graphical (above; bars with accompanying S.E.M.) and tabular (below; top row: gene name; bottom row: average proportionate expression ratio value) formats. The insert to each panel depicts relative ER variant and *CYP19A1* transcripts compared to *Ghrh* mRNA in young female rats; these data were mined from the data set from which information on *Ghrh* gene knockdown effect on absolute target gene expression in young female rats was previously reported [60].

Data shown in Fig. 6 (Ref. [60]) illustrate target gene versus *Ghrh* mRNA ratios in VMNdm Ghrh/SF-1 neurons from *SCR* siRNA/V (Fig. 6A), *Ghrh* siRNA/V (Fig. 6B), *SCR* siRNA/INS (Fig. 6C) and *Ghrh* siRNA/INS (Fig. 6D) old female rats. Like old males, euglycemic old female rats exhibit divergent target gene expression relative to *Ghrh*, with *GPER* mRNA expressed at the higher proportion. However, in young females, as in young males, *ERβ* is evidently the prevalent *ER* variant gene expressed in this neuron population. In VMNdm Ghrh neurons collected from old females, *Ghrh* gene silencing amplified each target gene profile relative to *Ghrh* and increased proportional *ERβ* expression (Fig. 6B). Young females, in contrast, exhibited down-regulated *ERα* and *GPER* mRNAs in response to this gene knockdown paradigm. Old hypoglycemic female rats exhibited increased relative *ERα* and *ERβ* gene transcription, which altered proportionate levels of these variants mRNAs versus *GPER* (Fig. 6C). In young animals, relative *ERα* and *GPER* gene expression was down-regulated due to hypoglycemia. Data in Fig. 6D indicate that in old and young hypoglycemic females, *Ghrh* gene knockdown substantially elevates the relative expression of each individual target gene. Results also show that VMN *Ghrh* gene silencing increased proportional *CYP19A1* gene transcription V- or INS-injected old and young female rats, and that in contrast to old male rats, hypoglycemia up-regulated this relative expression ratio in old females.

## 4. Discussion

Estradiol regulation of brain function requires insight into how normal- and patho-physiological processes affect brain cell sensitivity to this hormone signal. Aging is a natural, continuous process of change that affects all organ systems, including the brain, and purportedly alters ER variant gene and/or protein profiles in distinct brain regions or nuclei. The phenotypic and operational heterogeneity of cells that comprise these notable structures bolsters the need for continuing work to discern how aging affects ER expression in resident cell populations of known function. Current research illustrates the utility of multiplex qPCR technology, namely the capability for quantification of absolute and proportional expression profiles of diverse receptor targets for this hormone signal at the single-cell level, for research on the aging brain.

A Study has reported here examined how aging may affect *ER* variant gene expression in VMNdm Ghrh neurons appear to provide a complex, multi-modal neurochemical input to the counterregulatory neural network [60]. Present research also addressed the correlated premise that characteristic sex differences in ER transcription patterns in young animals may be likewise impacted by age. Results here document, for each sex, age-related absolute reductions in Ghrh neuron *ERα* and *GPER* gene expression levels, along with loss of *ERα* transcriptional reactivity to hypoglycemia. Effects of hypoglycemia on *ERβ* mRNA profiles varied between old male versus female rats, indicative of altered responsiveness in each sex due to age. The neuropeptide transmitter *Ghrh* regulates VMNdm Ghrh neuron ER variant transcript levels in young rats; data here document loss or change in direction of *Ghrh* control of distinctive *ER* mRNA profiles in old animals. Outcomes provide novel evidence that these neurons contain mRNA that encodes aromatase protein and that baseline expression of these transcripts is higher in old versus young rats of either sex, inferring that VMN Ghrh neuron neuroestradiol production may be enhanced with age, but cellular sensitivity to this local hormone signal may change during eu- and/or hypoglycemia. Current findings justify new efforts to ascertain how putative age-associated changes in absolute and proportionate signaling by ER variants may affect Ghrh neuron counterregulatory neurotransmission in each sex.

It is worthwhile considering that evidence for age-associated alterations in *ER* gene expression described here does not serve as conclusive confirmation that corresponding gene protein product production is likewise changed in a similar direction and magnitude. The plausible assumption that aging may elicit tandem adjustments *ER* gene versus protein expression under baseline and/or hypoglycemic conditions, will require application of proteomic analysis methods of requisite sensitivity for quantification of ER protein levels in single brain cell samples.

Administration of *Ghrh* siRNA to the old male rat VMN disclosed a loss of Ghrh neuromodulatory stimulation of VMNdm Ghrh nerve cell *ERα*, but not *ERβ* or *GPER* gene expression. Age-associated reductions in baseline *ERα*, but not *GPER* gene profiles in this sex may reflect Ghrh-dependent and -independent mechanisms, respectively. As *Ghrh* and *Ghrh* receptor mRNAs in this nerve cell type are respectively unaltered or decreased with age, attenuation of *Ghrh* control of *ERα* mRNA content may involve reduced *Ghrh* receptor activation of signaling pathways that regulate this *ER* gene profile. On the other hand, old female rats exhibit loss of *Ghrh* regulation of VMdm Ghrh *ERβ* and *GPER* gene profiles, but not *ERα* gene expression. As *Ghrh* and *Ghrh* receptor gene transcription are correspondingly diminished or refractory to aging, maintenance of *ERβ* gene profiles at levels observed in young animals may involve substitution of an alternative stimulus. Age-related down-regulated *ERα* and *GPER* gene expression in this sex may reflect loss of *Ghrh* positive tone in the former instance and age-associated emergence of a dominant inhibitory input in the latter scenario.

Co-expression of nuclear and membrane ER variants enables integrative estradiol regulation of VMNdm Ghrh neuron function [76–78]. It is well documented that the nuclear transcription factors *ERα* and *ERβ* play overlapping as well as unique roles in estradiol action *in vivo* as these variants exert dissimilar transcriptional activities in ligand-, cell type-, and promoter-specific contexts [79–81]. These ERs elicit nuclear or transcriptional effects by estrogen response element (ERE)-dependent and -independent mechanisms, which in the latter instance involves modulation of activities of other transcription factors such as activator protein-1, nuclear factor-kappa-B, and stimulating protein-1 [82]. Stimulation of membrane-anchored GPER causes cell type-specific rapid activation of signaling transduction cascades, namely phospholipase C/protein kinase, Ras/Raf/MAPK, phosphatidyl inositol 3 kinase/AKT, or cAMP/protein kinase A signaling cascades, which are capable of crosstalk with other signaling pathways [83–86]. Assimilation of extra-nuclear and nuclear estradiol regulatory effects will plausibly depend upon which signaling pathway(s) is/are activated and resulting phosphorylation of co-activators [87–90]. Fundamental questions that emerge from this and other recent work on VMNdm Ghrh neurons include the identity of GPER-activated signal transduction pathway(s) in these cells in young animals and potential effects of aging in each sex on pathway responsiveness to receptor stimulation.

Synchronized quantification of individual target gene profiles by multiplex qPCR analysis is a valuable tool for evaluation of relative expression of diverse *ER* transcripts within a common cellular source. Here, compilation of relative *ERα*, *ERβ*, and *GPER* mRNA expression ratios for VMNdm Ghrh neurons acquired from euglycemic, namely *SCR* siRNA-pretreated, V-injected old rats indicates that in each sex, *GPER* gene transcription in this nerve cell type is proportionately higher compared to *ERα* and *ERβ*. This indicates change from young adulthood where this nerve cell type is primarily subject to *ERβ*-mediated estradiol control. These data show that aging may significantly affect integration of estradiol input to VMNdm Ghrh nerve cells in each sex. *Ghrh* gene silencing here had profound sex-specific effects on mean *ER* variant gene expression ratios, as this genetic manipulation decreased proportional *GPER* gene expression in old males yet increased relative expression of all three *ER* variant mRNAs in the aged female while bringing *ERβ* and *GPER* expression ratios into closer alignment. These effects contrast with minimal effects of this treatment on relative *ER* variant gene expression in young animals of either sex. These results document an aging-associated gain in *Ghrh* inhibition of proportionate *GPER* gene expression (male) versus indiscriminate up-regulation of relative *ER* variant expression (female).

Data here show that IIH reduced proportionate *GPER* gene expression in old male rats, i.e., *SCR* siRNA/INS versus *SCR* siRNA/V treatment groups, without major effects on other *ER* variant mRNAs. This represents a change from young male rats, which exhibit pronounced down-regulated proportionate *ERα*, *ERβ*, and *GPER* gene expression. Thus, aging may cause a loss of hypoglycemic control of relative nuclear *ER* receptor gene transcription in this sex. *Ghrh* siRNA administration prior to hypoglycemia further reduced relative *GPER* gene expression in male rats. Results from old female rat VMNdm Ghrh neurons show that hypoglycemia elevated relative *ERα* and *ERβ* gene profiles, but did not alter the mean *GPER* expression ratio. Conversely, young INS-injected animals exhibit decreased proportionate *ERα* and *GPER* mRNA expression. *Ghrh* gene knockdown further augmented *ERα* and *ERβ* gene expression ratios and increased *GPER* relative expression compared to *SCR* siRNA-pretreated hypoglycemic old female rats. This pretreatment had similar effects on each *ER* variant relative expression in young female rats. Taken together, these results suggest that aging has differential effects on integrated estradiol regulation of VMNdm Ghrh neuron function during hypoglycemia, as relative *ER* variant gene expression is brought into alignment (as opposed to young animals) by reductions in proportional *GPER* gene expression in old males versus amplification of *ERα* and *ERβ* gene expression ratios in aged females.

The continuum of development over the male or female lifespan involves transition between distinct reproductive states, including reproductive senescence, which are typified by unique gonadal steroid hormone secretion patterns. Current findings show that VMNdm Ghrh neuron neuroestradiol production is also apparently age sensitive as *CYP19A1* gene profiles are enhanced in old versus young rats of either sex. As noted above, further experimentation is required to verify that aging amplifies CYP19A1 protein and enzyme activity profiles in this neuron population in each sex. Interestingly, eu- and hypoglycemic patterns of *CYP19A1* transcription are each up-regulated in response to VMN *Ghrh* gene silencing, which strongly suggests that this neuropeptide transmitter imposes a negative, inhibitory tone on this mRNA profile in each sex. The literature, as ably summarized by Cruz *et al*. [91], documents a progressive decline in female rat fertility between the ages of 8 to 12 months, characterized by reduced numbers of developing ovarian follicles and corpora lutea alongside increasing perturbances in estrous cyclicity, such as increased length, and development of constant estrous. Constant estrous, which occurs at 10 months and onward, is typified by low, unvarying circulating estradiol, estrone, testosterone, and progesterone levels, and an increase in estradiol/progesterone ratio. Female rats 12 months of age or older are reported to lack corpora lutea, a sign of absence of ovulation. While it is presumed that female subjects of similar age used in current work exhibited the attributes described above, the lack of direct verification of that supposition is a limitation. It is noted that potential changes in ovarian-derived estradiol signal volume to the aging female rat brain apparently coincide with possible augmentation of locally-generated neuroestradiol, as discussed above. Clearly, further studies are needed to quantify effects of age in both sex on net VMN tissue estradiol concentrations.

## 5. Conclusions

VMN is a known substrate for estradiol regulation of glucose homeostasis. Current work used combinative single-cell laser-catapult-microdissection/multiplex qPCR tools to establish if and how aging affects total and proportional VMN counterregulatory neuron *ER* variant gene expression in each sex. Results indicate that aging affects absolute *ERα* and *GPER*, but not *ERβ* mRNA levels in each sex, and abolished *ERα* (both sexes), *ERβ* (males), and *GPER* (males) transcriptional responses to hypoglycemia. Outcomes of VMN *Ghrh* gene silencing identify sex-specific age-related loss of neuropeptide control of total expression of discrete *ER* genes. Data also infer that aging likely affects integration of estradiol input to VMNdm Ghrh neurons during eu- and hypoglycemia. Euglycemic old animals, unlike young rats, exhibit higher relative baseline *GPER* gene transcription compared to *ERα* and *ERβ*. Moreover, with age, hypoglycemia causes sex-specific equalization of proportionate *ER* variant gene expression, as males show reductions in proportional *GPER* gene expression occur whereas *ERα* and *ERβ* relative expression ratios are increased in females. Current evidence for possible age-associated increases in VMNdm Ghrh neuroestradiol synthesis infers that autocrine stimulation of *ERβ* may be amplified by age. Additional effort is needed to investigate how age-associated modifications in absolute and proportionate signaling by distinctive ER may affect Ghrh neuron into to the neural counterregulatory network.

## Data Availability

The data that support the findings of this study are available from the corresponding author upon reasonable request.

## Abbreviations

CYP19A1: aromatase
ER*α*/ESR1: estrogen receptor-alpha
ER*β*/ESR2: estrogen receptor-beta
Ghrh: growth hormone-releasing hormone
Ghrh-R: Ghrh receptor
GPER: G protein-coupled estrogen receptor-1
IIH: insulin-induced hypoglycemia
INS: insulin
*sc*: subcutaneous
VMN: ventromedial hypothalamic nucleus
VMNdm: VMN dorsomedial division
